# (2-Chloro­phen­yl)(4-hy­droxy-1,1-dioxo-2*H*-1,2-benzothia­zin-3-yl)methanone

**DOI:** 10.1107/S1600536812014171

**Published:** 2012-04-06

**Authors:** Nazia Sattar, Hamid Latif Siddiqui, Matloob Ahmad, Waseeq Ahmad Siddiqui, Masood Parvez

**Affiliations:** a Institute of Chemistry, University of the Punjab, Lahore 54590, Pakistan; b Chemistry Department, Govt. College University, Faisalabad, Pakistan; c Chemistry Department, University of Sargodha, Sargodha 40100, Pakistan; d Department of Chemistry, The University of Calgary, 2500 University Drive NW, Calgary, Alberta, Canada T2N 1N4

## Abstract

In the title mol­ecule, C_15_H_10_ClNO_4_S, the heterocyclic thia­zine ring adopts a half-chair conformation, with the S and N atoms displaced by 0.527 (7) and 0.216 (7) Å, respectively, on opposite sides of the mean plane formed by the remaining ring atoms. The mol­ecular structure is consolidated by an intra­molecular O—H⋯O inter­action and the crystal packing is stabilized by N—H⋯O and C—H⋯O hydrogen bonds.

## Related literature

For background information on the synthesis of related compounds, see: Siddiqui *et al.* (2007 ▶). For the biological activity of 1,2-benzothia­zine derivatives, see: Ikeda *et al.* (1992 ▶); Lombardino *et al.* (1973 ▶); Gupta *et al.* (2002 ▶); Zia-ur-Rehman *et al.* (2006 ▶); Ahmad *et al.* (2010 ▶). For bromo analogue of the title compound, see: Sattar *et al.* (2012 ▶).
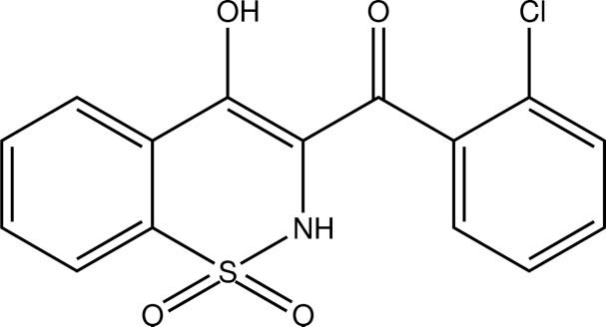


## Experimental

### 

#### Crystal data

C_15_H_10_ClNO_4_S
*M* *_r_* = 335.75
Monoclinic, 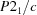

*a* = 12.1078 (6) Å
*b* = 8.4057 (5) Å
*c* = 14.7022 (9) Å
β = 105.541 (3)°
*V* = 1441.60 (14) Å^3^
*Z* = 4
Mo *K*α radiation
μ = 0.43 mm^−1^
*T* = 173 K
0.12 × 0.10 × 0.06 mm

#### Data collection

Nonius KappaCCD diffractometer
Absorption correction: multi-scan (*SORTAV*; Blessing, 1997 ▶) *T*_min_ = 0.951, *T*_max_ = 0.975
5606 measured reflections
3204 independent reflections
2383 reflections with *I* > 2σ(*I*)
*R*_int_ = 0.051

#### Refinement

*R*[*F* ^2^ > 2σ(*F* ^2^)] = 0.068
*wR*(*F* ^2^) = 0.138
*S* = 1.12
3204 reflections
203 parameters
H atoms treated by a mixture of independent and constrained refinement
Δρ_max_ = 0.36 e Å^−3^
Δρ_min_ = −0.36 e Å^−3^

### 

Data collection: *COLLECT* (Hooft, 1998 ▶); cell refinement: *DENZO* (Otwinowski & Minor, 1997 ▶); data reduction: *SCALEPACK* (Otwinowski & Minor, 1997 ▶); program(s) used to solve structure: *SHELXS97* (Sheldrick, 2008 ▶); program(s) used to refine structure: *SHELXL97* (Sheldrick, 2008 ▶); molecular graphics: *ORTEP-3 for Windows* (Farrugia, 1997 ▶); software used to prepare material for publication: *SHELXL97*.

## Supplementary Material

Crystal structure: contains datablock(s) global, I. DOI: 10.1107/S1600536812014171/zj2065sup1.cif

Structure factors: contains datablock(s) I. DOI: 10.1107/S1600536812014171/zj2065Isup2.hkl

Supplementary material file. DOI: 10.1107/S1600536812014171/zj2065Isup3.cml

Additional supplementary materials:  crystallographic information; 3D view; checkCIF report

## Figures and Tables

**Table 1:** Hydrogen-bond geometry (Å, °)

*D*—H⋯*A*	*D*—H	H⋯*A*	*D*⋯*A*	*D*—H⋯*A*
N1—H1*N*⋯O4^i^	0.84 (4)	2.06 (4)	2.857 (4)	158 (4)
C13—H13⋯O2^ii^	0.95	2.59	3.307 (5)	132
C5—H5⋯O1^iii^	0.95	2.55	3.369 (5)	145
O3—H3*O*⋯O4	0.84	1.80	2.536 (3)	146

Symmetry codes: (i) 
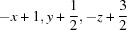
; (ii) 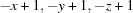; (iii) 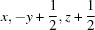.

**Fig. 1.**
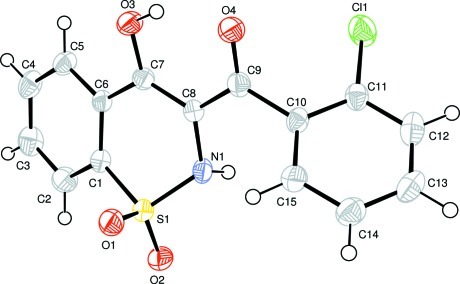
The molecular structure of the title compound with the atom numbering scheme. Displacement ellipsoids are drawn at the 50% probability level. H atoms are presented as small spheres of arbitrary radius.

**Fig. 2.**
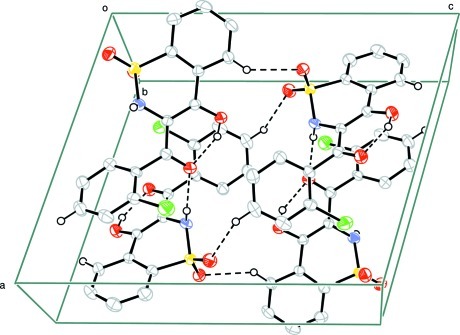
A part of the unit cell showing intermolecular and intramoilecular hydrogen bonds (dotted lines) in the crystal structure of the title compound. H atoms non-participating in hydrogen-bonding were omitted for clarity.

## supplementary crystallographic information

### Comment

The nucleus 1,2-benzothiazine 1,1-dioxide represents a class of pharmaceutically important heterocyclic compounds that have received considerable attention because of their dynamic structural features and a wide range of biological activities. They are known to be anti-allergy (Ikeda *et al.*, 1992), anti-inflammatory (Lombardino *et al.*, 1973), analgesic (Gupta *et al.*, 2002), anti-bacterial (Zia-ur-Rehman *et al.*, 2006) *etc*. In continuation of our research on the synthesis of biologically active benzothiazine derivatives (Siddiqui *et al.*, 2007; Ahmad *et al.*, 2010) we report herein the synthesis and crystal structure of the title compound.

The bond distances and angles in the title compound (Fig. 1) agree very well with the corresponding bond distances and angles reported for its bromo analogue with which it is isomorphic (Sattar *et al.*, 2012). The heterocyclic thiazine ring adopts a half chair conformation with atoms N1 and S1 displaced by 0.216 (7) and 0.527 (7) Å, respectively, on the opposite sides from the mean plane formed by the remaining ring atoms. The molecular structure is stabilized by intramolecular interactions O4–H4O···O3 and the crystal packing is consolidated by N1—H1N···O4 and C13—H13···O2 intermolecular hydrogen bonds (Figure 2 and Table 1).

### Experimental

A mixture of 2-[2-(*o*-chlorophenyl)-2-oxoethyl]-1,2-benzisothiazol-3(2*H*)-one 1,1-dioxide (10.1 g, 30 mmol) and sodium methoxide (2.2 g, 40.0 mmol) in freshly dried methanol (25 ml) was subjected to reflux for 30 minutes. The reaction was quenched with ice-cold water and acidified to pH = 3 with dilute HCl. The precipitates were filtered, washed with water and ethanol (25 ml, each) to get yellow powder of the title compound (9.4 g, 70%). The crystals suitable for X-ray crystallographic analysis were grown from a mixture of solvents chloroform and methanol (2:1) by slow evaporation at room temperature.

### Refinement

The H atoms bonded to C and O atoms were positioned geometrically and refined using a riding model, with O—H and C—H = 0.84 and 0.95 Å, respectively. The amino H-atom was allowed to refine freely. The *U*_iso_(H) were allowed at 1.2*U*_eq_(parent atom).

### Crystal data

**Table:**

C_15_H_10_ClNO_4_S	*F*(000) = 688
*M**_r_* = 335.75	*D*_x_ = 1.547 Mg m^−^^3^
Monoclinic, *P*2_1_/*c*	Mo *K*α radiation, λ = 0.71073 Å
Hall symbol: -P 2ybc	Cell parameters from 2930 reflections
*a* = 12.1078 (6) Å	θ = 1.0–27.5°
*b* = 8.4057 (5) Å	µ = 0.43 mm^−^^1^
*c* = 14.7022 (9) Å	*T* = 173 K
β = 105.541 (3)°	Prism, pale yellow
*V* = 1441.60 (14) Å^3^	0.12 × 0.10 × 0.06 mm
*Z* = 4	

### Data collection

**Table:**

Nonius KappaCCD diffractometer	3204 independent reflections
Radiation source: fine-focus sealed tube	2383 reflections with *I* > 2σ(*I*)
Graphite monochromator	*R*_int_ = 0.051
ω and φ scans	θ_max_ = 27.5°, θ_min_ = 2.9°
Absorption correction: multi-scan (*SORTAV*; Blessing, 1997)	*h* = −15→15
*T*_min_ = 0.951, *T*_max_ = 0.975	*k* = −10→10
5606 measured reflections	*l* = −19→19

### Refinement

**Table:**

Refinement on *F*^2^	Primary atom site location: structure-invariant direct methods
Least-squares matrix: full	Secondary atom site location: difference Fourier map
*R*[*F*^2^ > 2σ(*F*^2^)] = 0.068	Hydrogen site location: inferred from neighbouring sites
*wR*(*F*^2^) = 0.138	H atoms treated by a mixture of independent and constrained refinement
*S* = 1.12	*w* = 1/[σ^2^(*F*_o_^2^) + 4.364*P*] where *P* = (*F*_o_^2^ + 2*F*_c_^2^)/3
3204 reflections	(Δ/σ)_max_ < 0.001
203 parameters	Δρ_max_ = 0.36 e Å^−^^3^
0 restraints	Δρ_min_ = −0.36 e Å^−^^3^

### Special details

**Table:**

Geometry. All e.s.d.'s (except the e.s.d. in the dihedral angle between two l.s. planes) are estimated using the full covariance matrix. The cell e.s.d.'s are taken into account individually in the estimation of e.s.d.'s in distances, angles and torsion angles; correlations between e.s.d.'s in cell parameters are only used when they are defined by crystal symmetry. An approximate (isotropic) treatment of cell e.s.d.'s is used for estimating e.s.d.'s involving l.s. planes.
Refinement. Refinement of *F*^2^ against ALL reflections. The weighted *R*-factor *wR* and goodness of fit *S* are based on *F*^2^, conventional *R*-factors *R* are based on *F*, with *F* set to zero for negative *F*^2^. The threshold expression of *F*^2^ > σ(*F*^2^) is used only for calculating *R*-factors(gt) *etc*. and is not relevant to the choice of reflections for refinement. *R*-factors based on *F*^2^ are statistically about twice as large as those based on *F*, and *R*- factors based on ALL data will be even larger.

### Fractional atomic coordinates and isotropic or equivalent isotropic displacement parameters (Å^2^)

**Table:**

	*x*	*y*	*z*	*U*_iso_*/*U*_eq_	
Cl1	0.71096 (9)	0.38782 (15)	0.83022 (8)	0.0469 (3)	
S1	0.18808 (7)	0.39935 (12)	0.60671 (6)	0.0281 (2)	
O1	0.1528 (2)	0.2392 (3)	0.58127 (18)	0.0358 (7)	
O2	0.1631 (2)	0.5208 (4)	0.53640 (19)	0.0396 (7)	
O3	0.3300 (2)	0.2131 (3)	0.88114 (18)	0.0340 (6)	
H3O	0.3920	0.1661	0.8837	0.041*	
O4	0.5099 (2)	0.1471 (3)	0.83156 (18)	0.0341 (6)	
N1	0.3252 (2)	0.3988 (4)	0.6544 (2)	0.0278 (7)	
H1N	0.358 (3)	0.487 (5)	0.655 (3)	0.033*	
C1	0.1348 (3)	0.4509 (5)	0.7026 (3)	0.0273 (8)	
C2	0.0344 (3)	0.5377 (5)	0.6909 (3)	0.0386 (10)	
H2	−0.0038	0.5812	0.6312	0.046*	
C3	−0.0087 (4)	0.5595 (5)	0.7679 (3)	0.0411 (10)	
H3	−0.0778	0.6179	0.7605	0.049*	
C4	0.0460 (3)	0.4985 (5)	0.8555 (3)	0.0386 (10)	
H4	0.0147	0.5155	0.9074	0.046*	
C5	0.1461 (3)	0.4128 (5)	0.8679 (3)	0.0318 (8)	
H5	0.1832	0.3699	0.9281	0.038*	
C6	0.1931 (3)	0.3892 (4)	0.7913 (2)	0.0249 (7)	
C7	0.3004 (3)	0.3016 (4)	0.8035 (2)	0.0253 (7)	
C8	0.3659 (3)	0.3096 (4)	0.7403 (2)	0.0250 (7)	
C9	0.4744 (3)	0.2291 (5)	0.7586 (3)	0.0284 (8)	
C10	0.5468 (3)	0.2430 (4)	0.6910 (3)	0.0265 (8)	
C11	0.6580 (3)	0.3042 (5)	0.7190 (3)	0.0300 (8)	
C12	0.7263 (4)	0.3083 (5)	0.6570 (3)	0.0406 (10)	
H12	0.8011	0.3525	0.6765	0.049*	
C13	0.6856 (4)	0.2483 (5)	0.5672 (3)	0.0406 (10)	
H13	0.7326	0.2502	0.5248	0.049*	
C14	0.5764 (4)	0.1854 (5)	0.5387 (3)	0.0391 (10)	
H14	0.5490	0.1417	0.4772	0.047*	
C15	0.5063 (3)	0.1858 (5)	0.5996 (3)	0.0349 (9)	
H15	0.4301	0.1465	0.5785	0.042*	

### Atomic displacement parameters (Å^2^)

**Table:**

	*U*^11^	*U*^22^	*U*^33^	*U*^12^	*U*^13^	*U*^23^
Cl1	0.0364 (5)	0.0581 (7)	0.0450 (6)	−0.0087 (5)	0.0087 (4)	−0.0126 (5)
S1	0.0242 (4)	0.0341 (5)	0.0262 (4)	−0.0017 (4)	0.0072 (3)	0.0021 (4)
O1	0.0359 (14)	0.0401 (16)	0.0324 (14)	−0.0094 (13)	0.0109 (11)	−0.0065 (13)
O2	0.0331 (14)	0.0514 (19)	0.0334 (15)	0.0028 (14)	0.0073 (12)	0.0157 (14)
O3	0.0298 (14)	0.0426 (17)	0.0314 (14)	0.0113 (13)	0.0112 (11)	0.0104 (13)
O4	0.0292 (13)	0.0410 (17)	0.0329 (14)	0.0093 (12)	0.0096 (11)	0.0062 (13)
N1	0.0233 (15)	0.0292 (17)	0.0328 (17)	−0.0023 (14)	0.0106 (13)	0.0036 (14)
C1	0.0260 (17)	0.028 (2)	0.0296 (18)	−0.0022 (15)	0.0096 (14)	0.0008 (16)
C2	0.032 (2)	0.042 (2)	0.041 (2)	0.0089 (19)	0.0087 (17)	0.007 (2)
C3	0.034 (2)	0.039 (2)	0.054 (3)	0.0106 (19)	0.0177 (19)	0.000 (2)
C4	0.037 (2)	0.043 (2)	0.044 (2)	0.0059 (19)	0.0248 (19)	0.000 (2)
C5	0.0342 (19)	0.035 (2)	0.0291 (19)	0.0018 (18)	0.0140 (16)	0.0007 (17)
C6	0.0235 (16)	0.0253 (18)	0.0263 (17)	−0.0032 (15)	0.0075 (14)	−0.0001 (15)
C7	0.0248 (17)	0.0252 (19)	0.0257 (17)	0.0006 (15)	0.0067 (14)	0.0005 (15)
C8	0.0217 (16)	0.0281 (19)	0.0254 (17)	−0.0016 (15)	0.0063 (13)	0.0006 (15)
C9	0.0262 (18)	0.031 (2)	0.0279 (18)	−0.0024 (16)	0.0077 (14)	−0.0055 (16)
C10	0.0248 (17)	0.0269 (19)	0.0306 (18)	0.0054 (15)	0.0124 (14)	0.0016 (15)
C11	0.0272 (18)	0.027 (2)	0.037 (2)	0.0015 (16)	0.0111 (16)	0.0030 (16)
C12	0.033 (2)	0.039 (2)	0.055 (3)	0.0013 (19)	0.021 (2)	0.004 (2)
C13	0.047 (2)	0.040 (2)	0.043 (2)	0.008 (2)	0.027 (2)	0.008 (2)
C14	0.041 (2)	0.044 (3)	0.034 (2)	0.007 (2)	0.0143 (18)	0.0022 (19)
C15	0.031 (2)	0.042 (2)	0.033 (2)	0.0027 (18)	0.0104 (16)	0.0012 (18)

### Geometric parameters (Å, º)

**Table:**

Cl1—C11	1.737 (4)	C4—H4	0.9500
S1—O2	1.427 (3)	C5—C6	1.404 (5)
S1—O1	1.431 (3)	C5—H5	0.9500
S1—N1	1.620 (3)	C6—C7	1.462 (5)
S1—C1	1.755 (4)	C7—C8	1.376 (5)
O3—C7	1.329 (4)	C8—C9	1.438 (5)
O3—H3O	0.8400	C9—C10	1.497 (5)
O4—C9	1.250 (4)	C10—C15	1.387 (5)
N1—C8	1.437 (5)	C10—C11	1.395 (5)
N1—H1N	0.84 (4)	C11—C12	1.386 (5)
C1—C2	1.388 (5)	C12—C13	1.375 (6)
C1—C6	1.405 (5)	C12—H12	0.9500
C2—C3	1.380 (6)	C13—C14	1.381 (6)
C2—H2	0.9500	C13—H13	0.9500
C3—C4	1.379 (6)	C14—C15	1.388 (5)
C3—H3	0.9500	C14—H14	0.9500
C4—C5	1.379 (5)	C15—H15	0.9500
			
O2—S1—O1	119.57 (18)	O3—C7—C8	122.4 (3)
O2—S1—N1	108.01 (17)	O3—C7—C6	114.4 (3)
O1—S1—N1	108.05 (18)	C8—C7—C6	123.2 (3)
O2—S1—C1	110.69 (18)	C7—C8—N1	119.7 (3)
O1—S1—C1	107.08 (17)	C7—C8—C9	120.9 (3)
N1—S1—C1	102.01 (17)	N1—C8—C9	119.4 (3)
C7—O3—H3O	109.5	O4—C9—C8	120.5 (3)
C8—N1—S1	116.8 (2)	O4—C9—C10	119.1 (3)
C8—N1—H1N	114 (3)	C8—C9—C10	120.4 (3)
S1—N1—H1N	115 (3)	C15—C10—C11	118.5 (3)
C2—C1—C6	121.0 (3)	C15—C10—C9	119.9 (3)
C2—C1—S1	121.9 (3)	C11—C10—C9	121.5 (3)
C6—C1—S1	116.9 (3)	C12—C11—C10	120.9 (4)
C3—C2—C1	118.6 (4)	C12—C11—Cl1	118.3 (3)
C3—C2—H2	120.7	C10—C11—Cl1	120.7 (3)
C1—C2—H2	120.7	C13—C12—C11	119.9 (4)
C4—C3—C2	121.5 (4)	C13—C12—H12	120.0
C4—C3—H3	119.2	C11—C12—H12	120.0
C2—C3—H3	119.2	C12—C13—C14	120.0 (4)
C5—C4—C3	120.2 (4)	C12—C13—H13	120.0
C5—C4—H4	119.9	C14—C13—H13	120.0
C3—C4—H4	119.9	C13—C14—C15	120.3 (4)
C4—C5—C6	119.9 (4)	C13—C14—H14	119.9
C4—C5—H5	120.0	C15—C14—H14	119.9
C6—C5—H5	120.0	C10—C15—C14	120.4 (4)
C5—C6—C1	118.7 (3)	C10—C15—H15	119.8
C5—C6—C7	120.8 (3)	C14—C15—H15	119.8
C1—C6—C7	120.5 (3)		
			
O2—S1—N1—C8	166.9 (3)	C6—C7—C8—N1	−4.5 (6)
O1—S1—N1—C8	−62.4 (3)	O3—C7—C8—C9	−3.8 (6)
C1—S1—N1—C8	50.2 (3)	C6—C7—C8—C9	175.9 (3)
O2—S1—C1—C2	33.6 (4)	S1—N1—C8—C7	−34.2 (5)
O1—S1—C1—C2	−98.3 (4)	S1—N1—C8—C9	145.4 (3)
N1—S1—C1—C2	148.3 (3)	C7—C8—C9—O4	1.7 (6)
O2—S1—C1—C6	−151.4 (3)	N1—C8—C9—O4	−177.9 (3)
O1—S1—C1—C6	76.7 (3)	C7—C8—C9—C10	−178.0 (3)
N1—S1—C1—C6	−36.7 (3)	N1—C8—C9—C10	2.4 (5)
C6—C1—C2—C3	−1.5 (6)	O4—C9—C10—C15	117.4 (4)
S1—C1—C2—C3	173.3 (3)	C8—C9—C10—C15	−63.0 (5)
C1—C2—C3—C4	0.6 (7)	O4—C9—C10—C11	−58.7 (5)
C2—C3—C4—C5	−0.2 (7)	C8—C9—C10—C11	120.9 (4)
C3—C4—C5—C6	0.7 (6)	C15—C10—C11—C12	0.3 (6)
C4—C5—C6—C1	−1.6 (6)	C9—C10—C11—C12	176.4 (4)
C4—C5—C6—C7	178.7 (4)	C15—C10—C11—Cl1	176.4 (3)
C2—C1—C6—C5	2.0 (6)	C9—C10—C11—Cl1	−7.5 (5)
S1—C1—C6—C5	−173.0 (3)	C10—C11—C12—C13	−1.5 (6)
C2—C1—C6—C7	−178.4 (4)	Cl1—C11—C12—C13	−177.7 (3)
S1—C1—C6—C7	6.6 (5)	C11—C12—C13—C14	0.6 (7)
C5—C6—C7—O3	17.5 (5)	C12—C13—C14—C15	1.5 (7)
C1—C6—C7—O3	−162.2 (3)	C11—C10—C15—C14	1.8 (6)
C5—C6—C7—C8	−162.3 (4)	C9—C10—C15—C14	−174.4 (4)
C1—C6—C7—C8	18.0 (6)	C13—C14—C15—C10	−2.7 (6)
O3—C7—C8—N1	175.7 (3)		

### Hydrogen-bond geometry (Å, º)

**Table:**

*D*—H···*A*	*D*—H	H···*A*	*D*···*A*	*D*—H···*A*
N1—H1*N*···O4^i^	0.84 (4)	2.06 (4)	2.857 (4)	158 (4)
C13—H13···O2^ii^	0.95	2.59	3.307 (5)	132
C5—H5···O1^iii^	0.95	2.55	3.369 (5)	145
O3—H3*O*···O4	0.84	1.80	2.536 (3)	146

Symmetry codes: (i) −*x*+1, *y*+1/2, −*z*+3/2; (ii) −*x*+1, −*y*+1, −*z*+1; (iii) *x*, −*y*+1/2, *z*+1/2.
